# Added insult to injury? The response of meat-associated pathogens to proposed antimicrobial interventions

**DOI:** 10.1007/s00253-023-12849-x

**Published:** 2024-01-08

**Authors:** Maitiú Marmion, Guerrino Macori, Soukaina Barroug, Arturo B. Soro, Paula Bourke, Brijesh K. Tiwari, Paul Whyte, Amalia G. M. Scannell

**Affiliations:** 1 UCD School of Agriculture and Food Science, University College Dublin, Belfield, Dublin 4, D04V4W8 Ireland; 2 UCD Centre for Food Safety, University College Dublin, Belfield, Dublin 4, D04V4W8 Ireland; 3 UCD School of Biosystems and Engineering, School of Public Health, Physiotherapy & Sports Science, University College Dublin, Belfield, Dublin 4, D04V4W8 Ireland; 4 UCD School of Veterinary Medicine, University College Dublin, Belfield, Dublin 4, D04V4W8 Ireland; 5 Department of Food Chemistry and Technology, Teagasc Food Research Centre, Ashtown, Dublin 15, D15 KN3K Ireland; 6 UCD Institute of Food and Health, University College Dublin, Belfield, Dublin 4, D04V4W8 Ireland

**Keywords:** Antimicrobial, Stress response, *Salmonella*, Meat processing, *Campylobacter*

## Abstract

**Abstract:**

Modern requirements for ‘green label’ meat products have led to the design of novel antimicrobial innovations which prioritise quality, safety and longevity. Plasma-functionalised water (PFW), ultraviolet light and natural antimicrobial compositions have been investigated and optimised for control of foodborne pathogens like *Campylobacter jejuni* and *Salmonella enterica* serovar Typhimurium. However, given the adaptive mechanisms present in bacteria under external stresses, it is imperative to understand the effect that sublethal treatment may have on the bacterial transcriptome. In this study, *Salmonella* Typhimurium and *C. jejuni* were treated with sublethal doses of ultraviolet light, a citrus juice/essential oil marinade, and ‘spark’ or ‘glow’ cold plasma generation system-produced PFW. Immediately after treatment, cells were lysed and RNA was extracted and purified. mRNA was converted to cDNA by reverse transcription-PCR and sequenced by an Illumina MiSeq® system. Sequences were filtered and analysed using the Tuxedo workflow. Sublethal treatment of *Campylobacter jejuni* and *Salmonella* Typhimurium led to increased immediate cellular and metabolic activity, as well as diversification in protein and metabolic functioning. There was further expression of pathogenesis and virulence-associated traits associated with spark PFW and marinade treatment of *Salmonella* Typhimurium*.* However, similar concerns were not raised with glow PFW or UV-treated samples. This study provides science-based evidence of the efficacy of multi-hurdle antimicrobial system using green-label marinades and PFW or UV to inactivate pathogens without upregulating virulence traits in surviving cells. This study will inform policymakers and food industry stakeholders and reinforces the need to incorporate in-line novel technologies to ensure consumer safety.

**Key points:**

• *Salmonella and C. jejuni showed increased cell activity in immediate response to stress.*

• *Virulence genes showed increased expression when treated with natural antimicrobials and sPFW.*

• *Reduced immediate transcriptomic response to gPFW and UV treatment indicates lower risk.*

**Supplementary Information:**

The online version contains supplementary material available at 10.1007/s00253-023-12849-x.

## Introduction

Chicken meat has become the primary animal protein source in many regions of the world, with its low-cost, high-producibility and nutritious benefits being key reasons for growth in its consumption (Organisation for Economic Co-operation and Development 2022). However, poultry meat is frequently associated with human illness caused by bacterial pathogens including *Campylobacter* and *Salmonella*. These pathogens may be dispersed during processing, contaminating end products, equipment and surfaces (Rasschaert et al. 2020). Though steps such as washing and chilling are employed to control pathogen proliferation, these steps are not effective at removing pathogen burdens to levels below those recommended by regulatory bodies such as the European Food Safety Authority (EFSA) and the United States Food and Drug Administration (USFDA) With up to 90% of retail chicken meat contaminated with at least one of these pathogens as well as millions of suspected cases of campylobacteriosis and salmonellosis occurring annually, it has become imperative to design and implement antimicrobial interventions which can effectively control these pathogens (Golden and Mishra 2020; Marmion et al. 2021).

In order to be deemed acceptable, antimicrobial interventions for products intended for human consumption must be accepted by regulatory bodies and consumers (Korzen et al. 2011; MacRitchie et al. 2014). Chemical washes of poultry carcasses, for example, are limited in certain jurisdictions, while consumer hesitancy around irradiative approaches limits their use in fresh food (Stefani et al. 2014; Bearth and Siegrist 2019). As such, new interventions which have high efficacy, low cost, low invasiveness, and minimal impact on product quality are deemed to be desirable by manufacturers and consumers alike.

Ultraviolet light is light within the spectrum of wavelengths between 200 and 400 nm. Light within the UV-C (200–280 nm) range can have a particular effect on bacterial cells, damaging DNA and genetic materials and structures leading to cell inactivation and death (Lu et al. 2019; Soro et al. 2020). Furthermore, UV light treatments can lead to the production of oxidative species, causing further cell injury and death (Kebbi et al. 2020). This approach offers benefits such as the possibility of in-package treatment, low invasiveness, effectiveness against pathogens, and declining cost as light-emitting diode (LED) UV equipment becomes more prevalent and efficient (Kebbi et al. 2020; Soro et al. 2021a).

Cold plasma is created through the passage of a current through a gas medium, leading to an ionised gas which can create free radical species which can inactivate microbiological contamination on exposed surfaces, including food products (Yepez et al. 2022). These radical species can be created and stored in deionised water through application of atmospheric plasma to the water, creating a compound known as plasma-functionalised water (PFW). PFW contains superoxide or hydroxide radicals, depending on the plasma system in use, which in turn create reactive oxygens or reactive nitrogen species (ROS, RNS) which have potent antimicrobial effects against microbes (Thirumdas et al. 2018; Barroug et al. 2021). These approaches can be employed in-package as well as incorporated into scald tank or washing steps, as well as environmental and direct product treatment, which gives potential applications for cold plasma and PFW in the food industry.

Interventions within domestic settings to limit bacteria growth are often confined to hygiene practices, effective cooking, maintaining packaging integrity, and cold storage. These steps however can be ineffective, with many cases of sporadic meat-related food poisoning traced back to home preparation of meat (Tack et al. 2019; Ray et al. 2021). Further issues such as reduced shelf-life and spoilage are also linked to domestic storage and preparation practices. To circumvent these issues, marinades with both a flavouring and antimicrobial function were designed for use on poultry meat; the process for this is described in Marmion et al. (2023) (unpublished). These marinades utilised effective antimicrobial concentrations of essential oils, citrus juices and herbs which are known to cause stress to several bacterial structures and functions including membranes, protein folding and general metabolic function (Ozogul et al. 2015; Pedrós-Garrido et al. 2020; Marmion et al. 2022a). Green-label marinades have the advantage of being both possible value-added products and employable in the home, causing reductions in bacterial populations without compromising consumer fears about ‘unnatural’ interventions against foodborne disease.

While these technologies have been found to be effective both *in vitro* and on meat surfaces, there are justifiable concerns regarding bacterial responses to these stresses when the effect is sublethal, possibly leading to adaptation in surviving bacterial cells. External and internal stresses are known to cause changes in the transcriptome of cells which increase tolerance which can have downstream effects for how bacteria act when consumed by humans. Factors such as environmental acidification are known to induce the expression of pathogenicity gene cassettes in non-typhoidal *Salmonella enterica* serovars, while ROS can induce the expression of MacAB-type efflux systems in *Salmonella* which can lead to its survival against ROS-based antimicrobials as well as increase cell virulence (de Jong et al. 2012; Bogomolnaya et al. 2020; Marmion et al. 2022b). Furthermore, it has been found that *Campylobacter* stress tolerance is linked to exposure to and survival of previous stressors. This includes acid stress, desiccation and starvation tolerance which are encountered within the production chain (Kim et al. 2021). Therefore, as this preconditioning may be provided by incomplete antimicrobial steps, it is essential to assess whether these treatments can increase the risk posed by surviving pathogens after treatment, given the potential for altered virulence, conditional resilience and antimicrobial tolerance of bacteria which have been exposed to stresses.

Two poultry-associated pathogens were grown to log phase and treated with sublethal variations of treatments which incorporate the approaches listed above. Once cells were treated, the antimicrobial effect was quenched as appropriate, and RNA was extracted from lysed cells. RNA was converted to cDNA via reverse transcription polymerase sequencing approaches which were employed to assess changes in transcriptome of treated cells under sublethal stress.

This study aimed to investigate the transcriptomic response of *S. enterica* serovar Typhimurium and *C. jejuni* to spark-treated PFW, glow-treated PFW, ultraviolet LED light, and an antimicrobial marinade consisting of lemon juice, thyme oil and black pepper.

## Materials and methods

### Strains and growth conditions

*Campylobacter jejuni* NCTC11168 was grown overnight in Bolton broth supplemented with defibrinated horse blood and Bolton broth growth supplement (Oxoid Media, Basingstoke, UK) at 37 °C. This overnight suspension was used to inoculate biphasic media within a tissue culture flask (*Brucella* agar base overlaid with brain-heart infusion (BHI) broth containing 1 % defibrinated horse blood) at a ratio of 1:100. The bacterium was allowed to grow with gentle shaking to mid-log phase growth at 37 °C before being centrifuged at 5000 × *g* utilising a Thermo Scientific Heraeus Megafuge 16R Centrifuge (Heraeus, Hanau, Germany) and washing with sterile ¼ strength Ringer diluent. These cells were then pelleted at 5000 × *g* prior to treatment with the antimicrobial intervention of interest and subsequent extraction of RNA.

*Salmonella enterica* ser. Typhimurium ATCC14028 was grown overnight in tryptic soy broth (TSB). This overnight suspension was used to inoculate brain-heart infusion broth containing 1 % defibrinated horse blood at a ratio of 1:100. This was allowed to grow to mid-log phase growth at 37 °C before being centrifuged at 5000 × *g* and washing with sterile ¼ strength Ringer diluent. These cells were then pelleted at 5000 × *g* prior to treatment with the antimicrobial intervention of interest and the extraction of RNA.

### Cell treatment and RNA extraction

Three forms of novel antimicrobial methods were chosen for assessment in this report: antibacterial marinades, ultraviolet LED light, and cold plasma PFW. The prepared cell suspensions were treated with antimicrobial interventions as described below. Once cells were treated, RNA was extracted as described in the QiAgen® RNEasy MicroRNA extraction kit. RNA was subjected to overnight ethanol extraction as described in Koolman et al. (2016) and resuspended in RNase-free water and quantified using a Thermo Fisher® NanoDrop 1000 (Thermo Scientific, Wilmington, DE, USA). RNA was stored in a RNase-free Eppendorf tube at −20 °C until RNA sequencing could be completed. An untreated mid-log phase control for each species was also included in this protocol.

#### Marinades

Marinades based on those designed in Marmion et al. (2023) (unpublished) were selected based on their antimicrobial efficacy. A marinade composed of 0.5% (v/v) thyme oil in lemon juice with 1% (w/v) black pepper was made and was diluted by a factor of two with sterile deionised water to reduce the lethal stress applied to bacterial cells. Cells were grown to mid-log phase as described above and pelleted in a 50-ml Falcon tube at 5000 × *g* for 10 min. This pellet was resuspended in the dilute marinade composition to achieve a shock effect and immediately pelleted again at 5000 × *g* for 3 min. The supernatant was immediately discarded, and cells were washed and resuspended twice in a phosphate-buffered saline (PBS) solution. After the second wash step, cells were pelleted as described above prior to RNA extraction with the QiAgen RNeasy isolation kit.

#### Plasma

functionalised water. Plasma-functionalised water (PFW) was generated using two different plasma set-ups namely spark and glow using the reactive species specificity (RSS) plasma system (School of Biosystem and Food Engineering, UCD). This system consisted of a stainless needle employing a variable frequency of 20 to 65 kHz to treat deionised water for 15 min at a distance of 5 mm from the system needle tip by a spark or glow setting. For more information of the system employed, please consult Lu et al. (2017). This was done immediately prior to PFW use in this study. This treatment time was selected as it showed a lower lethal effect. Cells were treated with undiluted PFW as outlined in the protocol above, with 1 X Ringer solution used in the wash step instead of PBS. After the wash step, cells were pelleted as described above prior to RNA extraction with the QiAgen RNeasy isolation kit.

#### Ultraviolet LED

Ultraviolet light was employed to treat cells according to a protocol outlined by (Soro et al. 2021b). The pelleted cells were resuspended in sterile brain-heart infusion broth supplemented with 1% (v/v) defibrinated blood prior to UV treatment. The bacterial suspension was transferred to a sterile polystyrene petri dish and treated with 280-nm UV light for 6 min at a fluence rate of 0.041 W/cm^2^ to give a dosage of 0.246 W × min × cm^-2^ (dosage = fluence rate × treatment time). Treated cells were washed twice with sterile ¼ strength Ringer solution, and cells were pelleted as described above prior to RNA extraction with the QiAgen RNeasy isolation kit.

### Reverse transcription–polymerase chain reaction and sample barcoding

Reverse transcription–polymerase chain reaction (RT-PCR) was completed prior to Illumina MiSeq sequencing. In brief, RNA extracted from treated cell suspensions was quantified using an Invitrogen Qubit™ 2.0 Fluorometer (Invitrogen, Waltham, MA, USA) and a Qubit™ RNA High Sensitivity Assay. Once RNA concentration was established, 200 ng of bacterial RNA in nuclease-free water was reverse transcribed to cDNA according to the Oxford Nanopore cDNA-PCR sequencing kit protocol (SQK-PCS111). The length and quantity of the cDNA obtained were assessed using Agilent® TapeStation 4200 (Agilent, Santa Clara, CA, USA) and Thermo Fisher® NanoDrop 2.0 respectively.

The cDNA library was prepared according to a modified version of the Cut&Run library preparation protocol, using the NEBNext Ultra II DNA Library Prep Reaction Kit (NEB E7645) (New England Biolabs (NEB), UK) (Liu 2021). In brief, NEB adaptors were attached to the cDNA after an End Prep PCR step. Amplicons were selected according to size using AMPure XP beads and indexed according to an NEBNext Ultra II Q5 Index protocol (NEB Multiplex Oligos for Illumina Dual Index Primers Set 2, UK), and, after a second AMPure XP size selection step, final libraries were analysed with a Qubit Fluorometer and a Qubit™ DNA High Sensitivity Assay kit and an Agilent® Bioanalyzer. The ligation of barcodes was confirmed by qPCR and the NEBNext® Library Quant Kit for Illumina®.

This denatured library was prepared according to Illumina MiSeq kit instructions, utilising a PhiX library control (1%). Barcoded cDNA libraries pooled concentrations of 4 nM and 15 pM were denatured and loaded into a 150-cycle V3 flow cell and sequenced with Illumina MiSeq (Hayward, CA, USA) according to manufacturer instructions.

### Bioinformatic analysis

**Fig. 1:**
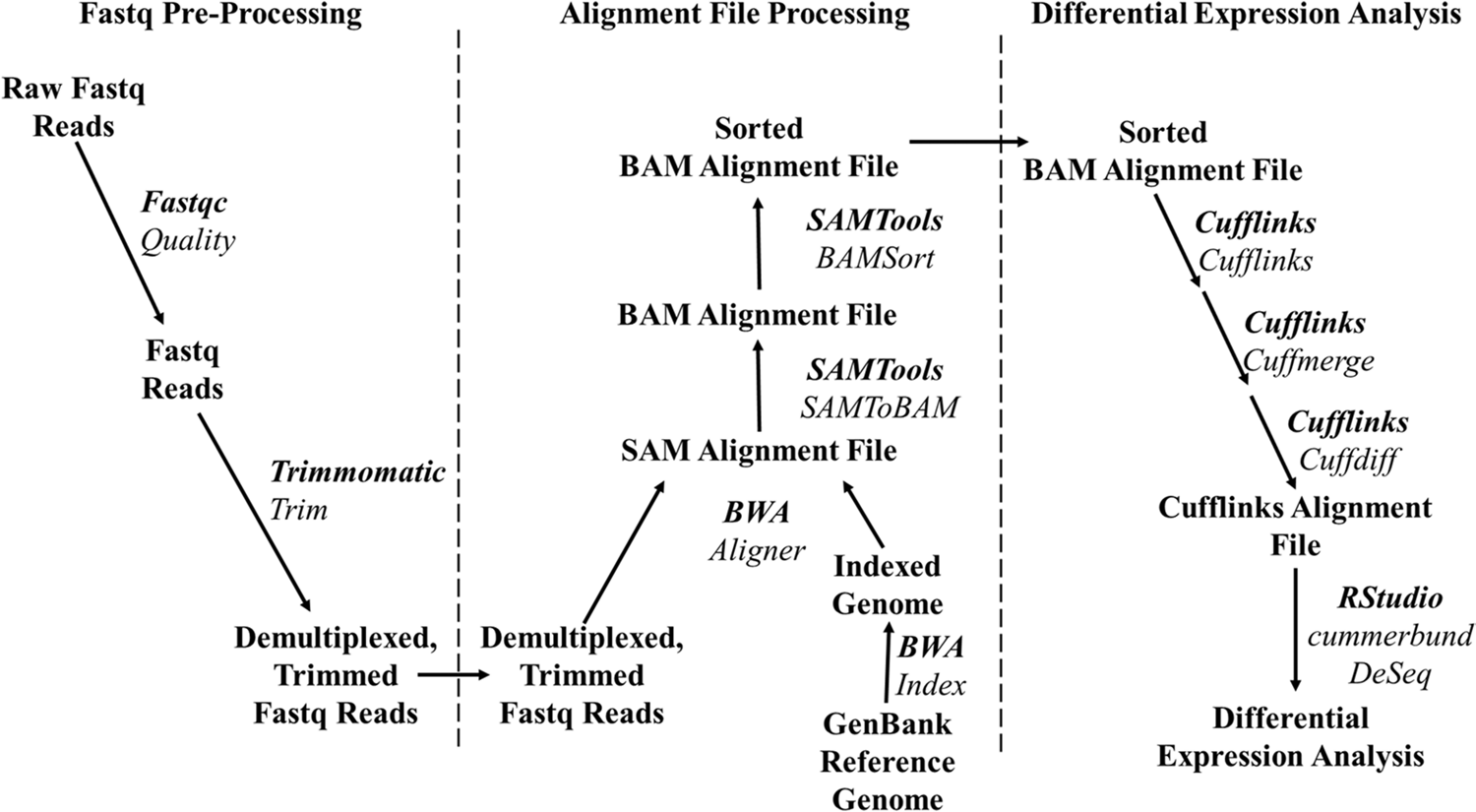
Bioinformatic steps followed for differential expression analysis

Once paired end fastq files were obtained, differential expression analysis was conducted on aligned annotated genomes (Fig. 1). 42,494,360 raw sequencing reads were obtained and analysed by fastqc and then processed as appropriate using Trimmomatic (Andrews 2010; Bolger et al. 2014). Sequence data is available on the NCBI sequence repository at http://www.ncbi.nlm.nih.gov/bioproject/973029. Once fastq files were processed and assigned appropriate sample labels, the Burrows Wheeler Aligner (BWA) tool was employed to align trimmed fastq reads to a *Salmonella enterica* serovar Typhimurium ATCC 14028 genome (GenBank CP102669.1) or *Campylobacter jejuni* NCTC11186 genome (GenBank NC_002163.1) obtained from NCBI GenBank (Li and Durbin 2009). The SAMTools programme was then used to convert and sort the obtained .sam alignment files to ordered .bam files which could be further analysed using Cufflinks software (Trapnell et al. 2010; Trapnell et al. 2012a). Cufflinks software and the CuffDiff function were used to index and assess differential expression patterns between samples. RStudio cummeRbund package software was then utilised for statistical and differential analysis (Trapnell et al. 2012b; Goff et al. 2022). Further analysis on gene ontology lists was conducted using the Panther software suite on the GeneOntology.org. resource centre (Mi et al. 2019; Thomas et al. 2022). This software suite allowed Fischer’s exact testing of metabolic and biological activity changes, utilising Bonferroni’s correction for more conservative analysis at a significance level *P* = 0.05.

## Results

**Fig. 2:**
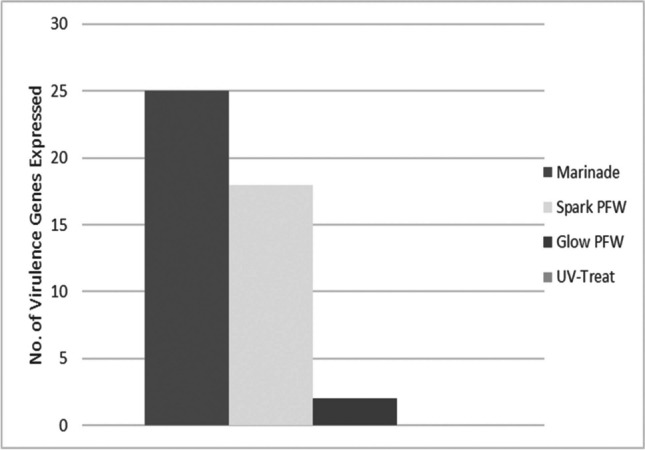
Immediate virulence gene expression in treated *Salmonella* Typhimurium (Suez et al. 2013)

**Fig. 3:**
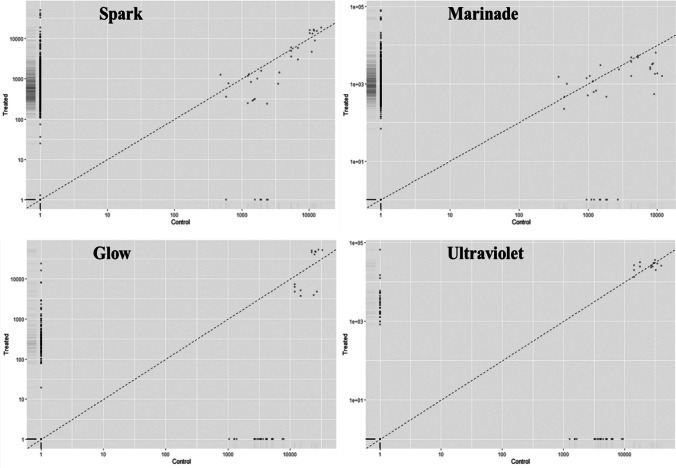
Gene expression similarity patterns in *Salmonella enterica* treated with sublethal novel antimicrobial interventions vs. control cultures

Expression patterns in treated *Salmonella enterica* ser. Typhimurium showed distinct changes in the transcriptional behaviour of cells from untreated control cultures when exposed to exogenous sublethal stress (Table S1). Cells changed their transcriptional patterns to adjust to the induction of stresses to cell structures and functions. Treated cultures tended to show an increase in cell activity immediately following treatment with the novel interventions, as well as a diversification in the pathways and biological processes induced in response to these stresses. Many virulence genes also saw increased expression, particularly in response to exposure to the antimicrobial marinade and spark-treated PFW (sPFW) (Fig. 2). Jensen-Shannon distance analysis, in which larger values reflect more disparate gene ontology patterns, showed distinct changes in the character of expressed groups of genes between control and treated *Salmonella*, particularly in cultures treated with the antimicrobial marinade and sPFW (Table 1). This is reinforced by the spatially distinct gene groupings seen in cummeRbund analysis of gene expression patterns (Fig. 3). It is notable that lower gene expression abundance and expression differences were seen in UV-treated *Salmonella*.

**Table 1:** Jensen-Shannon distance values between control and treated samples and *Salmonella enterica* treated with glow or spark cold plasma-treated plasma-functionalised water in terms of FPKM (fragment reads per kilobase of exon per million reads mapped) and TSS (transcriptional start sites)

Treatment	Jensen-Shannon distance	
	*Gene FPKM*	*TSS*
Glow	0.524601	0.5247101
Marinade	1.432418	1.452623
Spark	1.382325	1.404894
Ultraviolet light	0.5252653	0.5252653
Glow vs. spark	0.8807375	0.8825832

### *Campylobacter jejuni* response assessment

Results with treated and control *Campylobacter jejuni* showed low numbers of transcript recovery, with most transcripts from samples encoding genes involved in ribosome synthesis such as the 16S and 23S rRNA genes in both treated and untreated samples. This issue was compounded by high similarity between the transcript groups recovered, with Jensen-Shannon distance values of 0.23–0.37 recorded between control and treated samples. Treated samples tended to show an increase, though not significant (*P* > 0.05), in the expression of *Cj1637c*, *aroC*, *rnc*, and *rnhA*, which play roles in cell function and the processing of RNA, but further work would need to be completed to determine if this is statistically notable. Similarly, samples treated with UV light showed increased arginine tRNA production, which may be of interest given the photosensitivity of *Campylobacter* spp. An interesting gene which was noted to have increased expression after exposure to the antimicrobial marinade was *aas*, a gene encoding a 2-acylglycerophosphoethanolamine acyltransferase/acyl-acyl carrier protein synthetase which may play a role in broiler gastrointestinal tract (GIT) colonisation by motile *C. jejuni* variants (Hendrixson and DiRita 2004). This upregulation may relate to exposure to acidic conditions or citric acid derivatives which are present in both the host GIT and the citrus juice-based marinade. Similarly, *rnhA*, which is associated with a locus upregulated with treatment by PFW, is a ribonuclease associated with gallbladder colonisation in host animals. However, the results of this study do not necessarily represent a concern for consumers when *C. jejuni* is treated by these interventions, especially given the low recovery of transcript results. Given that *Salmonella* is regarded as a more pressing concern in the minds of consumers and health professionals, as well as more resilient when challenged by the interventions under investigation, the remainder of this report shall deal with this pathogen (Rothrock et al. 2017; Soro et al. 2021a).

### Treatment with antimicrobial compounds

**Fig. 4:**
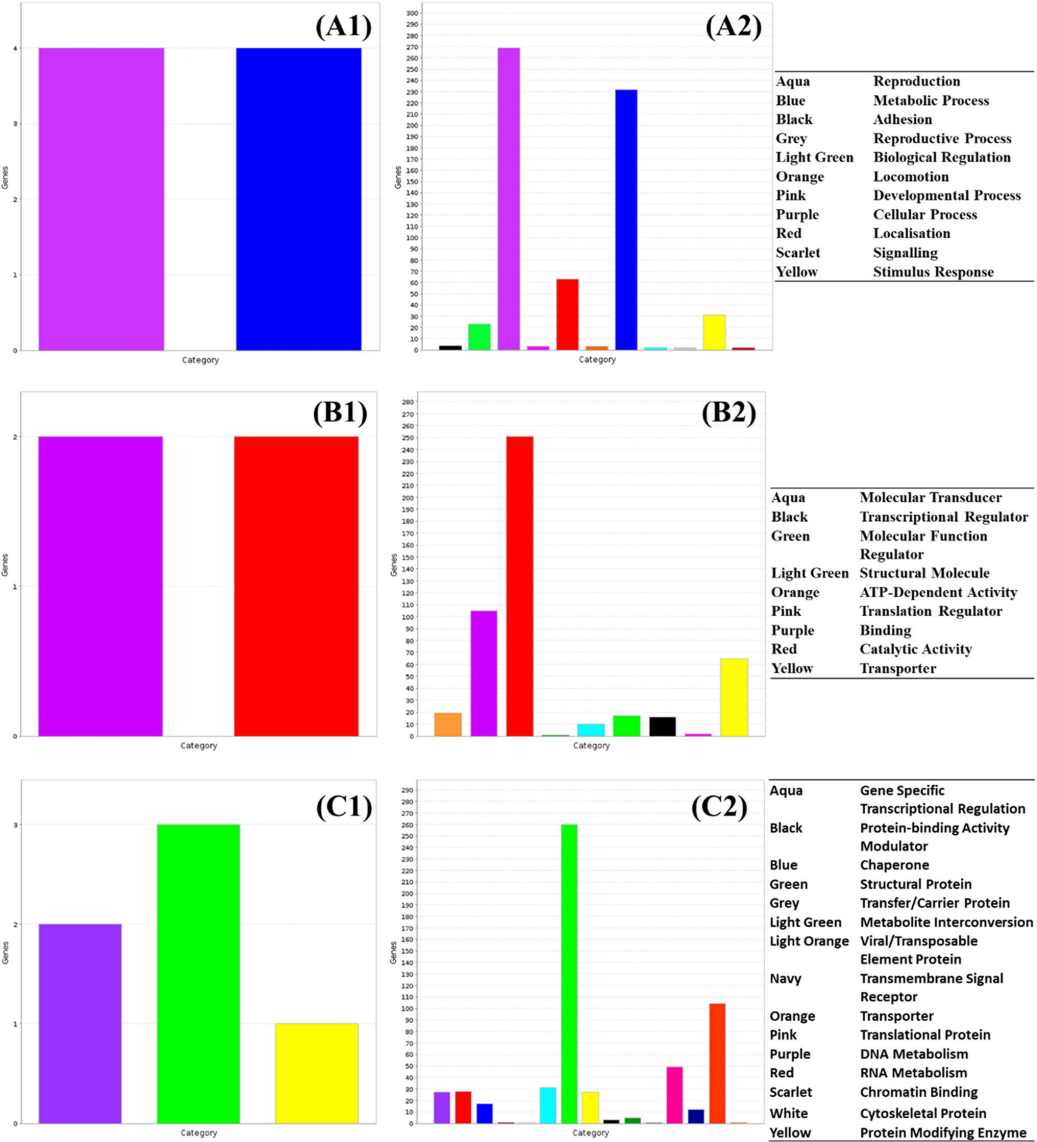
Change in relative expression of genes in (1) control and (2) antimicrobial marinade-treated *Salmonella enterica* ser. Typhimurium by **A** biological pathway, **B** molecular function and **C** protein function

Effective antibacterial marinades containing ingredients including essential oils and citrus juice were designed and optimised in a previous study (Marmion et al., 2023). The components of these marinades were intended to exploit cellular targets including the cell membrane (thyme essential oil) and metabolic function (citrus juice acidification). Treatment of *Salmonella* with these compounds led to an increase in cell function as more biological pathways, molecular functions and protein functions display relatively higher activity in treated cells (Fig. 4). In particular, cell and metabolic processes, catalytic activity and transporter function showed increased activity relative to the control culture, while specific transcriptional regulation proteins showed increased transcriptional activity under treated conditions.

Given the multifaceted stresses applied through these marinades, it is hoped that treated *Salmonella* serovars cannot adapt to this lethal stress and cannot reproduce to cause harm to consumers. On a metabolic level, there was a significant change (*P* < 0.05) in the synthesis of tRNAs, purine, glutamine, amino acids and pyruvate when *Salmonella* was treated with sublethal marinade concentrations. There were also notable but not significant (*P* > 0.05) changes in the expression of other metabolic functions and pathways, as well as efflux and export characteristics, biofilm components, and negative regulators of cell death pathways as the cell tries to negate the external stress posed by the marinade components.

Acid stress responses were noted in marinade-treated cells, including induction of the OmpR-EnvZ response and genes such as the succinate hydrogenase operon *sdhACD*, which is associated with acid tolerance, and *cadA*, which plays a role in cytoplasm neutralisation during environmental acidification (Ren et al. 2015; Kenney 2019). Similarly, there was downregulation of organic acid-producing metabolic processes in marinade-treated cells, which is in agreement with other studies of the *Salmonella* response to cytosolic acidification (Ren et al. 2015)*.*

However, it is also known that acid and membrane stress can trigger adaptations in target cells such as membrane modifications, initiation of efflux capabilities, metabolic modifications, and priming for survival of further stresses. Furthermore, it is known that acid stress is associated with the induction of *Salmonella* virulence characteristics within host organisms which could increase the threat posed (Chakraborty et al. 2015; Kenney 2019).

For example, the envelope-stress response sigma factor RpoE is relatively upregulated in treated *Salmonella*, which reflects the stress that the essential oil and acid components of the marinade have on treated membranes. This sigma factor tends to induce other upregulated genes such as the *rseABC* operon and genes involved in lipopolysaccharide synthesis (Amar et al. 2018). Envelope stress is also associated with phage shock response induction, although this response was not seen in this study as the *psp* operon repressor *pspA* was expressed in high abundance in treated samples (Darwin 2013; DeAngelis et al. 2019). However, PspA can function to stabilise membrane damage and maintain proton motive force gradients, as well as play a role in transmembrane transport. The results of this transcriptomic study reflect a more concerning trend however, as *pspA* is also a virulence-associated transcript (Karlinsey et al. 2010). Several virulence-associated traits were comparatively upregulated in the transcriptome of marinade-treated cells, including *hilC* and *hilD* which are key regulators in the initiation of the *Salmonella* pathogenicity island (SPI)-1 gene cassette, as well as effectors and proteins from SPI-1 (e.g. *sipA*, *prgH*, *sopA*, *sopB*, *sopD*) and SPI-2 (e.g. *sspH2*, *slrP*). Further putative virulence factors which are upregulated with marinade treatment such as the tripartite MdsABC efflux pump also play a role in oxidative stress response and antimicrobial resistance phenotypes (Song et al. 2015). The results of this study reflect other findings in cell responses to external stresses such as EO treatments with respect to the exogenous stress response protein *clpB*, though similar results were not seen with respect to *luxS*, *htpG*, *usp*, or *grpE* transcripts (Barbosa et al. 2020). It is notable that the ClpB chaperone which is induced in these conditions also plays a role in *Salmonella* type 6 secretion system effector export and virulence, underlining the putative role that incomplete treatment might play in the preconditioning pathogens for human infection.

### Treatment with cold plasma/plasma-functionalised water

gPFW and sPFW can vary in their effect on *Salmonella* cells, given the difference in stress posed by reactive nitrogen species (RNS) produced by gPFW or reactive oxygen species (ROS) produced by sPFW. ROS and RNS can target DNA, proteins/enzymes, membranes and amino acids, leading to biomolecule oxidation/peroxidation, membrane rupture due to electrostatic tension, peptidoglycan intramolecular bond breakage, lipooligosaccharide modification, and cell morphology changes (Misra et al. 2013). These stresses can cause cell death, but necessitate detoxification through different means (Alhasawi et al. 2019).

#### Spark PFW

**Fig. 5:**
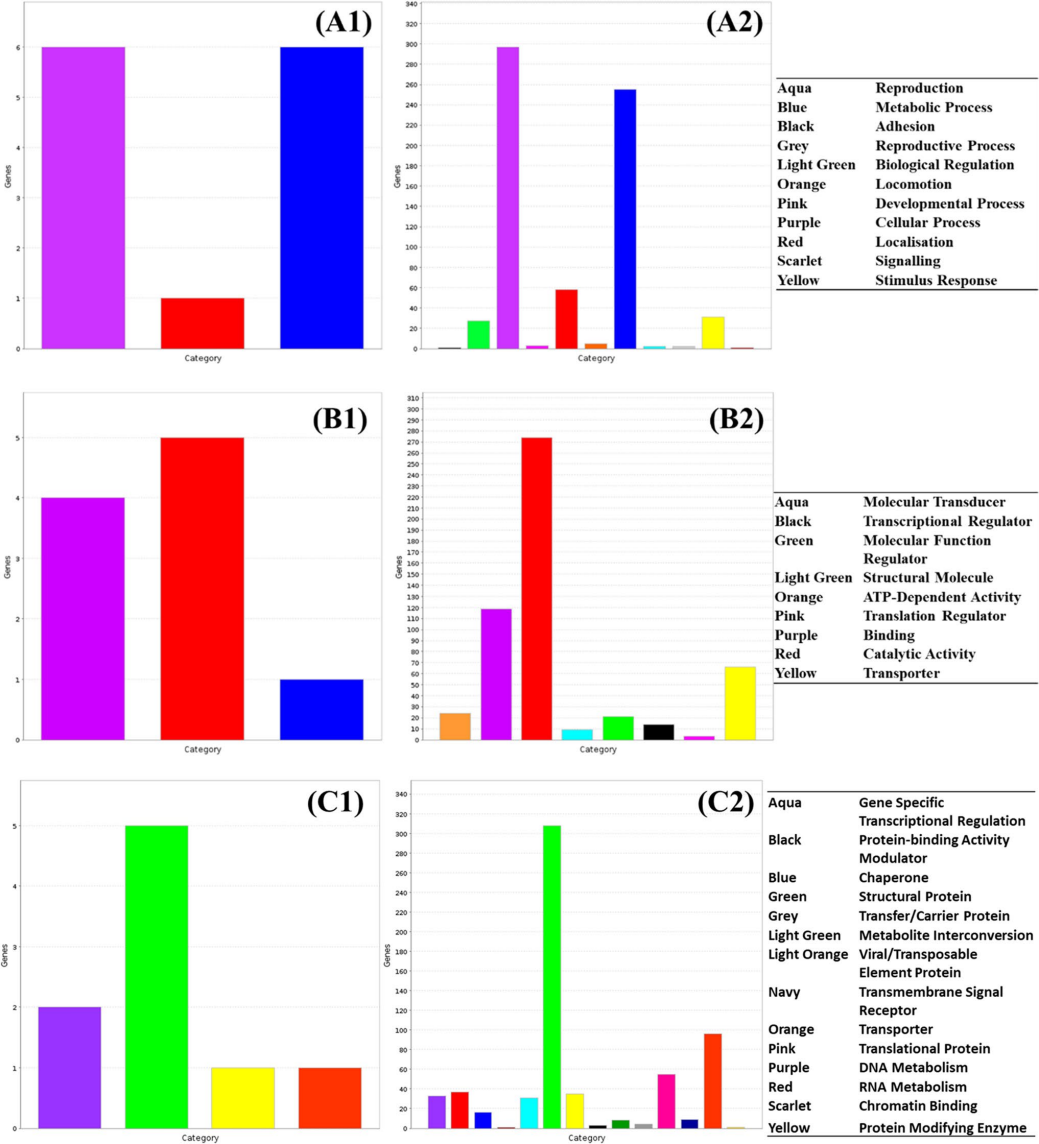
Change in relative expression of genes in (1) control and (2) spark PFW-treated *Salmonella enterica* ser. Typhimurium by **A** biological pathway, **B** molecular function and **C** protein function

Both gPFW and sPFW treatment caused an increase in both the activity and diversity of functions active within treated cells, with similar trends in increased metabolic, catabolic and cellular activities observed when compared to untreated cells (Figs. 4 and 5). Several more categories of proteins were induced in sPFW-treated *Salmonella* when compared to the control populations, with 14 protein categories induced to a relatively increased extent after treatment when compared to the control population, including RNA metabolism, translation, transport and gene specific transcription (Fig. 5).

sPFW treatment leads to significantly increased transcription of genes involved in several metabolic functions (*P* < 0.05), including tRNA synthesis, translation, protein and amino acid synthesis, and glucose metabolism, reinforcing the idea that the cell is increasing in metabolic and cellular activity to adapt to the changing environment. Similarly, other genes undergoing increased transcription in response to sPFW treatment include those involved in peroxide and other ROS response, nitrosative stress response, lipid biosynthesis, DNA damage, and further metabolic pathways. There was also downregulation of genes involved in biofilm formation.

As was the case with pathogen exposure to the antimicrobial marinade, treatment with sPFW caused an increase in both cell activity and diversification of cell activities with possible downstream consequences for food processors and consumers. Oxidative stress is a common means of controlling bacterial growth, with cells inducing genes/proteins such as catalases (e.g. *katE*, *katG*) and superoxide dismutases (e.g. *sodB*) in response to ROS related to exposure to peroxide exposure, for example, or sPFW treatment (Marmion et al. 2022a). Similar to other investigations assessing the effect of cold plasma interventions *on Salmonella*, sPFW induced the expression of the *oxyR* gene, which is vital in the mitigation of oxidative stresses in *Salmonella* (Cheng et al. 2020). Treatment with sPFW induced these oxidative stress response genes, as well as stress response sigma factors like *rpoS* and *rpoH* which allow broad stress-adaptive changes in the cell transcriptome including the envelope stress protective Rcs response and metabolic alterations, as well as DNA damage responsive transcriptomic mechanisms like *alkB* (Àlvarez et al. 2010).

The induction of *rpoH* in response to this stress raises similar concerns to the marinade treatment, given its role as a positive virulence regulator in *Salmonella* (Gruzdev et al. 2012). Further virulence-related genes found to be increasingly transcribed after treatment with sPFW included *hilC* and *hilD* which are involved in induction of SPI-1 transcription, a key part of host cell invasion, as well as SPI-1 and 2 effectors such as *sifA* and the *Salmonella* adhesin *siiE*. Similarly, there are concerns about the expression of antimicrobial resistance traits by treated cells such as the *mdsABC* efflux system and possible aminoglycoside resistance genes like *aac* (6’) which showed some upregulation after treatment with sPFW (Song et al. 2015; Mangat et al. 2017). The increased prevalence of virulence-related transcripts as well as resistance and resilience traits in treated cells represents some concerns about cell preconditioning during the ineffective use of sPFW as an antimicrobial intervention against *Salmonella* contamination.

#### Glow PFW

**Fig. 6:**
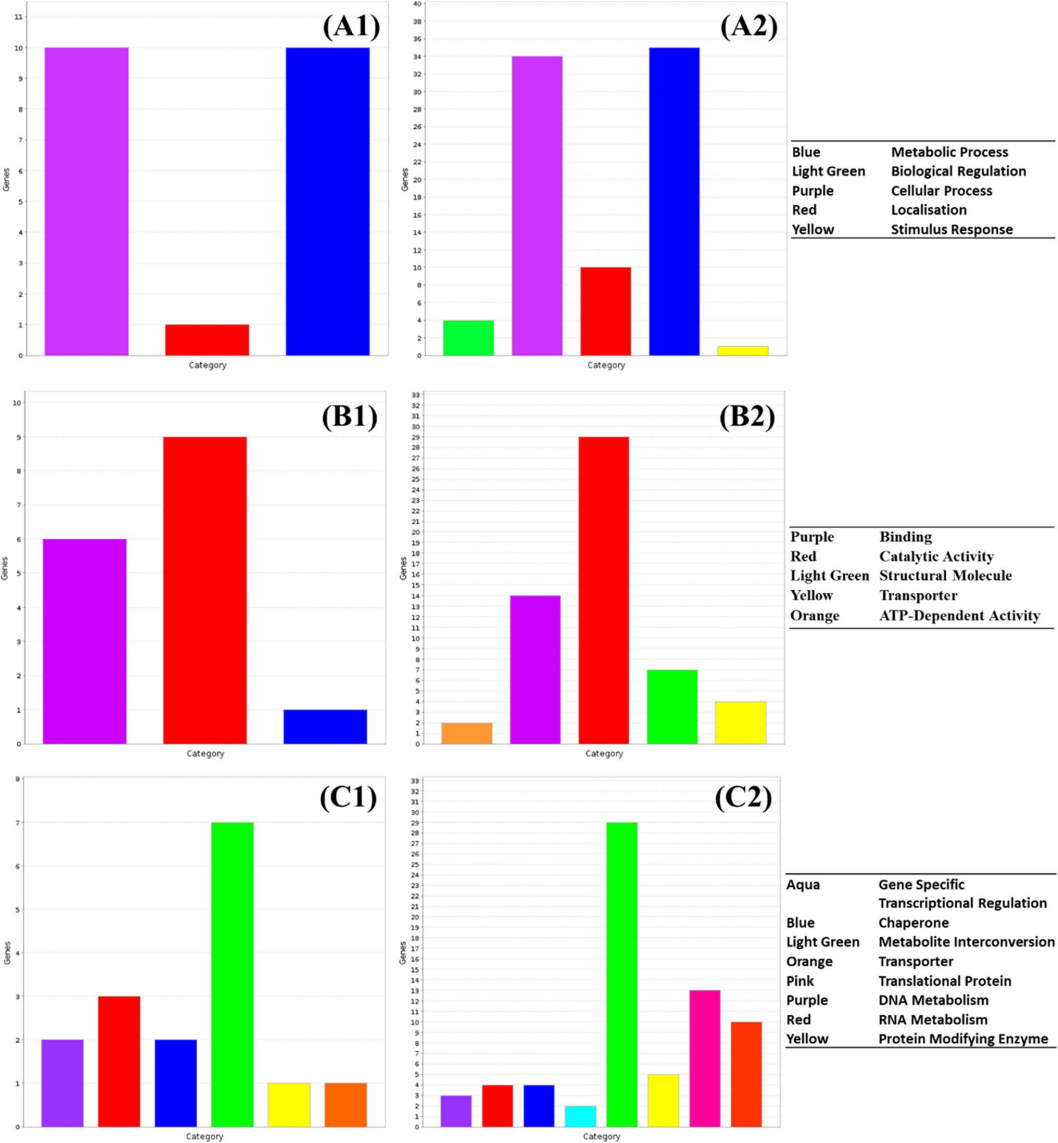
Change in relative expression of genes in (1) control and (2) glow PFW-treated *Salmonella enterica* ser. Typhimurium by **A** biological pathway, **B** molecular function and **C** protein function

Glow-treated PFW (gPFW) treatment of *Salmonella* cultures led to an increase in cell activity as well as an increase in the variety of cell processes underway. This included biological pathways involved in metabolic and cellular processes, as well as catalytic activities, metabolic interconversion, and translation components (Fig. 6). Many more genes were relatively more expressed in treated *Salmonella* cultures, including proteins involved in specific transcriptional regulation patterns and transport functions which were not reported to the same extent in the control culture.

On a transcriptional level, there were significant increases (*P* < 0.05) in the transcription of translation and peptide synthesis-related genes in treated *Salmonella* when compared to the control, while there was a significant decrease in nucleobase metabolism-related transcription. There were further notable, but not significant, increases in the transcription of genes related to the regulation of cell growth, DNA replication, protein synthesis and modification, and stress responses. However, there was also a small decrease in the expression of genes related to the oxidative stress response, which may relate to the different reactive species produced during gPFW synthesis. The large *Salmonella* adhesin *siiE* was one of the few virulence-related transcripts upregulated in response to gPFW treatment.

#### A comparison of *Salmonella* response to spark or glow PFW

**Fig. 7:**
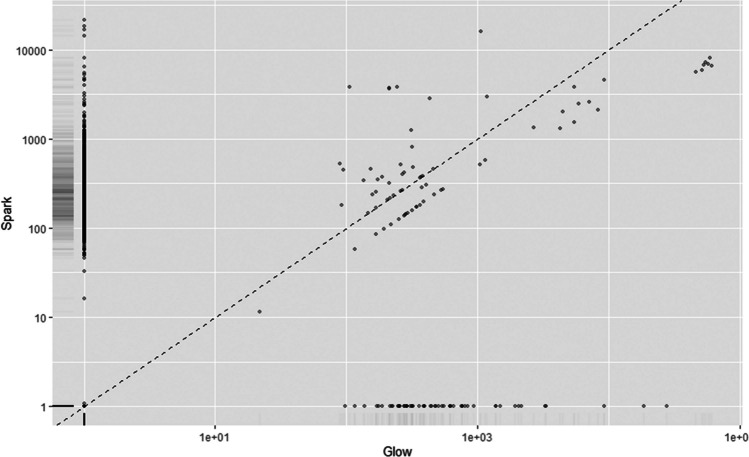
Gene expression patterns in *Salmonella enterica* treated with spark or glow cold plasma-treated plasma-functionalised water

A further direct comparison of the effect of sPFW and gPFW on *Salmonella* transcriptomics was conducted due to the difference in reactive radicals produced by these approaches. There were less concerns about possible pathogen preconditioning for virulence and survival relating to gPFW treatment than sPFW treatment. Where sPFW treatment led to the induction of several virulence and resistance traits as noted above, gPFW induced much fewer genes related to virulence or antimicrobial virulence while also inducing the virulence repressor gene *csrA* (Teplitski et al. 2006; Pannuri et al. 2016). While some similarity in transcriptional patterns was noted (Fig. 7), ontological analysis and Jensen-Shannon distance values indicated some differences in cell response to exogenous PFW-related stress (Table 1). Cold plasma treatment of water leads to the production of ROS or RNS within PFW which have broad but differing antimicrobial effects on cell structures and functions. This array of stresses and antimicrobial reactive species was shown to challenge the cells in different ways, as distinct patterns in sPFW and gPFW-treated cultures emerged.

Exploring this more, the different trends in relative expression were clearest in the increased cellular and metabolic activity of *Salmonella* treated with sPFW rather than gPFW. More diverse cell function was also noted, with an increase in the expression of proteins involved in processes such as transcription, translation and transport in cells treated with sPFW. Significant upregulation (*P* < 0.05) of transcript expression involved in the biosynthesis of nucleotides, sugars, amino acids, carrier molecules and tRNAs was seen in sPFW-treated cultures when compared to gPFW. However, there were also significantly increased (*P* < 0.05) expression of transcripts relating to DNA conformation and protein/amine metabolism in gPFW-treated cultures. These differences, as well as the clear increase in the expression of virulence characteristics in sPFW-treated *Salmonella*, underline the difference that the type of stress can play in the response of treated cells to stress, as well as possible concerns regarding the incomplete treatment of products with these antimicrobial interventions.

### Treatment with ultraviolet light

**Fig. 8:**
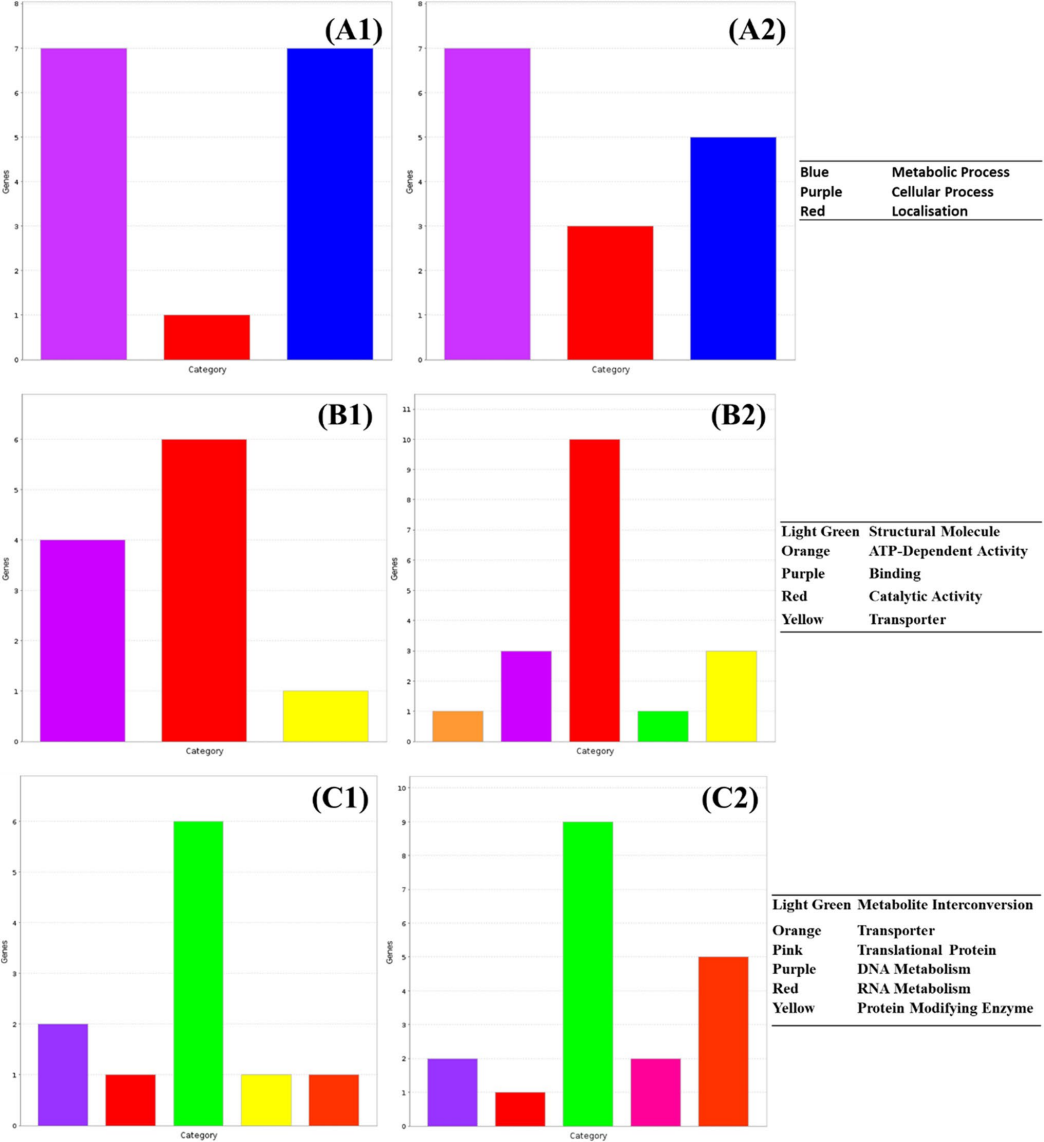
Change in relative expression of genes in (1) control and (2) UV-treated *Salmonella enterica* ser. Typhimurium by **A** biological pathway, **B** molecular function and **C** protein function

Ultraviolet light has been widely studied for its use as an antibacterial intervention in medical and industrial setting, with studies noting that Gram-negative species such as *Salmonella* showing increased vulnerability to UV treatment compared to Gram-positive bacteria (Soro et al. 2020). *Salmonella* cultures treated with UV light showed similar biological and protein expression patterns to control cultures (Fig. 8A, C). There was, however, a slight increase in the variety of significantly expressed molecular functions in UV-treated *Salmonella*, with treated *Salmonella* showing more catalytic, structural molecule function, and ATP-dependent activities than the control culture (Fig. 8B).

It is notable that no ontological gene groups were significantly increased or decreased in the treated culture when compared to the untreated one (*P* > 0.05). However, there were changes noted in the expression of genes associated with protein movement, translation, DNA conformation, metabolism, with treatment, while the control group showed higher expression of genes associated with carbohydrate and biomolecule and metabolism as well as DNA replication. The character of DNA management in treated vs. untreated *Salmonella* is notable given the role that DNA damage plays in the antimicrobial function of UV light, with the DNA relaxing topoisomerase IV upregulated in UV-treated cells (Aldred et al. 2013). This upregulation may allow the tolerance of UV-linked mutagenic crosslinking through DNA relaxation, though further studies would be needed to investigate this thoroughly. The increased production of some chaperone proteins was also noted, which may aid in the protection of the misfolded proteins. However, questions regarding the effectiveness of transcriptomic studies with UV damage response can be raised given the disruptive effect that DNA damage related to UV treatment has on cell transcription, with lower abundances of cDNA transcripts isolated from UV-treated cells than other treatments employed in this study (McKay et al. 2004). Some DNA damage responses such as the alkylation response *tag* and *ogt* genes thus tend to be constitutively expressed in the cytoplasm and might be better assessed in proteomic-based studies (Àlvarez et al. 2010).

## Discussion

Novel antibacterial interventions in food production favour low invasiveness, low sensorial remnants, efficient energy use, consumer acceptability and safety, and high antimicrobial efficacy. These criteria have led to the development of interventions that can be implemented within the processing line, such as wash modifications utilising chemicals or plasma-functionalised water, direct treatment with cold plasma and modified chilling/freezing steps, in-package steps such as modified packaging gases, cold plasma, high-pressure processing and ultraviolet light, and post-packaging/in-home interventions such as chilling and antibacterial marination steps (Loretz et al. 2010; Kalchayanand et al. 2022; Blandon et al. 2023). Many of these steps have been researched in depth and established as possible interventions for both improving product shelf-life and enhancing food safety. However, it is well known that sublethal extracellular stresses can lead to alterations in cell resilience and virulence as the bacterium becomes preadapted to future stress, while certain antimicrobial challenges such as chemical stress may be associated with antibiotic resistance traits (Akbar and Anal 2013; Liao et al. 2018).

A further consideration based upon the current study is that it assessed only the initial transcriptomic response of *Salmonella enterica* ser. Typhimurium when exposed to the antibacterial stresses. Therefore, further work is needed to investigate the effect of exposure over time. This concern is evident with some target genes like stress-responsive sigma factors like σS and σE which were not as highly induced as might be expected. However, similar temporally divergent transcriptomic responses have been reported in related species such as cold-stressed *E. coli* Sakai, in which immediate temporary, immediate sustained, and delayed transcriptomic responses to stress were recorded (King et al. 2016; Mitosch et al. 2019). Furthermore, bacterial response to stress is not wholly transcription-based, with other systems such as toxin-antitoxin systems, mRNA degradative pathways, and anti-sigma factors playing roles in the maintenance of cell response to external conditions (Wu et al. 2019; Vargas-Blanco and Shell 2020; Marmion et al. 2022b; Smith et al. 2023). The response of *Salmonella* to the novel stresses utilised in this report may or may not be maintained over time as cells adapt to the challenge posed. This would require further investigation given the intended duration for which these stresses employed. For example, washing with PFW or UV light may be a relatively short-term exposure, while marination tends to be a longer process, potentially allowing cells to adapt effectively. This may have knock-on effects for consumers, especially as the relative proportion of possibly vulnerable consumers increases as the global population gets older (Lund 2015).

The high burden of bacterial foodborne disease remains a challenge for meat processors. Food companies and researchers have investigated the efficacy of potential novel risk mitigation interventions as plasma-functionalised water, ultraviolet light and natural antimicrobials which have shown strong antimicrobial effect against relevant pathogens in challenge tests. However, bacteria have consistently shown the ability to adapt to exogenous stress and may remain an issue after treatment. The transcriptional patterns of treated cultures showed that these concerns may be valid in the immediate aftermath of treatment, as *Salmonella enterica* showed some upregulation of virulence and stress tolerance-associated factors such as pathogenicity island genes in response to these interventions. In particular spark created PFW, and the antimicrobial marinade composition invoked these responses of concern. Similarly, *Campylobacter jejuni* showed a small increase in the expression of colonisation-related genes under exogenous stress. These findings reinforce the importance in ensuring that optimal use of these interventions is designed and implemented by researchers and industry partners, with the end goal of a safer end product for the consumer.

## Supplementary information

ESM 1 (PDF 245 kb)

## Data Availability

Data is available on the NCBI sequence repository at http://www.ncbi.nlm.nih.gov/bioproject/973029.
