# Supplementary material for: Added insult to injury? The response of meat-associated pathogens to proposed antimicrobial interventions
Source: Appl Microbiol Biotechnol. 2024 Jan 8;108(1):87. doi: 10.1007/s00253-023-12849-x (PMC10774175; doi:10.1007/s00253-023-12849-x)
Supplement: Supplementary file 1 — (PDF 245 kb) [file 253_2023_12849_MOESM1_ESM.pdf]

Supplementary Tables

Journal : Applied Microbiology and Biotechnology

Title: Added Insult to Injury? The Response of Meat-Associated Pathogens to Proposed Antimicrobial Interventions

Authors: [1,4] Marmion, Maitiú; [1,4] Macori, Guerrino; [5] Barroug, Soukaina; [2, 6] Soro, Arturo B.; [5] Bourke, Paula; [6] Tiwari, B. K.; [2]Whyte, Paul; [1, 3, 4] Scannell, Amalia G. M

Affiliations: [1]UCD School of Agriculture and Food Science, [2]UCD School of Veterinary Medicine, [3]UCD Institute of Food and Health, [4]UCD Centre for Food Safety, [5]UCD School of Biosystems and Engineering, School of Public Health, Physiotherapy & Sports Science; University College Dublin, Belfield, Dublin 4, Ireland, D04V4W8. [6] Department of Food Chemistry and Technology, Teagasc Food Research Centre, Ashtown, Dublin 15, D15 KN3K, Ireland.

Corresponding author: Maitiú Marmion; matthew.marmion@ucdconnect.ie

Supplementary Table 1. *Genes upregulated in Control and Treated Salmonella enterica ser. Typhimurium.*

**Treatment**	**Control Samples**	**Treated Samples**
Antimicrobial Marinade	**30 Genes/Loci:** *araB, ccmA, ccmB, fdhE, fdoI, fdxH, groL, NUW86_00545, NUW86_00875, NUW86_02255, NUW86_02265, NUW86_05725, NUW86_05735, NUW86_08340, NUW86_15095, NUW86_19225, NUW86_19240, NUW86_21055, NUW86_22615, NUW86_22625, NUW86_22830, NUW86_22845, NUW86_23550, NUW86_24050, ppnN, rho, rseP, tcuA, tcuB, wecA*	**1675 Genes/Loci:** *accA, accD, aceA, aceE, aceF, acnA, acnB, acrB, acrD, acs, adeP, adhE, adk, alaC, alaS, allR, amiB, amtB, amyA, apbC, araA, arcA, arcA, arcB, argB, argE, argG, argP, argR, argS, arnA, arnC, arnD, arnE, arnF, arnT, aroC, aroG, artJ, artM, artQ, artP, asnA, asnB, aspS, atpA, atpB, atpD, atpG, atpH, bamA, barA, bcsE, bcsF, bcsG, bglX, bioA, bioB, bioC, bioD, bioF, birA, murB, brxL, btsT, btuB, murI, btuF, mtnN, cadA, caiF, carA, carB, casA, casB, cdsH, cgtA, chbG, cheW, cheZ, chiP, citD, citE, citS, clpB, clpX, cls, cmoM, mukB, mukE, mukF, coaBC, dut, copA, corA, corC, cpxA, cpxR, cra, creB, creC, crr, csgD, cstA, cyaA, cybC, cycA, cydA, cydB, cydC, cydD, cysA, cysP, cysT, cysW, cysD, cysG, cysI, cysJ, cysM, cysS, dacA, dacC, dacD, damX, dapA, dapD, dcd, dctA, dcuA, ddlA, deaD, yrbN, der, dgcJ, dgcQ, dgt, dinB, dksA, dld, dnaJ, dnaK, dnaX, dpiA, dpiB, dppB, dppC, dppD, dppF, dprA, smg, dsdX, dtpB, dusB, dxs, eamA, edd, emrB, eno, entD, entS, envC, envZ, ompR, epd, epmB, eptA, pmrA, ettA, eutA, eutH, eutK, eutP, eutQ, eutT, pta, fabB, fabF, fabG, fabI, fabY, fadA, fadB, fadI, fadJ, fbp, fdnG, fdnG, fepB, fepC, fepD, fepG, fetA, fetB, fhuE, fimH, fis, fldB, flgK, fliB, fliF, fliG, fliH, fliI, fnr, folK, pcnB, folP, glmM, foxA, frdA, frdB, fruA, fruB, fruK, fsa, ftsE, ftsX, ftsI, mraY, murE, murF, ftsL, rsmH, ftsW, murD, murG, ftsY, ftsZ, fucP, fumA, fusA, gabP, gabT, galK, galM, galP, gapA, gcl, gcvP, ggt, ghxP, glgA, glgC, glgP, glk, glmS, glmU, glnD, glnE, glnP, glnQ, glnS, glpK, glpX, glrR, qseG, gltB, gltD, gltJ, gltK, gltL, gltP, glyQ, glyS, gntR, gntT, gph, rpe, trpS, gpmA, grcA, greA, gsiA, gspE, hofC, gss, guaB, gudD, gyrA, gyrB, hcp, helD, hemA, hemB, hflC, hflK, hflX, hilD, hisA, hisB, hisC, hisD, hisF, hisH, hisIE, hisP, hmsP, hns, holA, lptE, hpaA, hpaE, hpaG, hpaX, hrpA, hscA, hsdR, hslU, hypB, hypC, hypD, hypE, iaaA, iadA, iagB, ibpA, ibpB, icd, ileS, lspA, ilvA, ilvC, ilvG, ilvM, infB, infC, invC, spaK, spaM, spaN, spaO, spaP, iolE, iolG1, iolH, iolI2, iprA, iscR, iscS, kdgA, kdpB, kdpC, kdpD, kdpE, kdsB, ycaR, kefB, kefG, kefC, kefF, kup, lapB, ldtB, lepB, leuA, leuB, leuS, lexA, lgt, lhgO, ligA, lipA, livF, livG, livH, livM, livJ, lolC, lolD, lolE, lon, lpdA, lpfE, lplA, lpoB, thiK, lptB, lptD, lptF, lptG, lpxA, lpxB, rnhB, lpxC, lpxD, lpxM, lpxO, lrp, lysA, lysP, lysS, macA, macB, maeB, maiA, malK, malM, malP, malZ, mazG, mdsA, mdsB, mdtA, mdtB, mdtK, mdtQ, melB, metB, metH, metK, metL, metQ, mfd, mgtA, miaA, mutL, mlrA, mnmE, mnmG, mpl, mrcA, mrcB, mrdB, mreB, mreC, mscM, mscS, mutM, nadD, nagB, nagK, nagZ, nanA, nanT, napA, napD, napF, narK, nfi, nfsB, nhaR, nifJ, nirB,*

		nirD, nlpI, norV, norW, npr, rapZ, nrdB, yfaE, nrdD, nrfA, nudC, nudE, nudK, nuoC, nuoE, nuoF, nuoJ, nuoK, nuoL, nuoM, nuoN, nupC, nusA, NUW86_00030, NUW86_00050, NUW86_00055, dgoD, dgoR, NUW86_00115, NUW86_00130, NUW86_00140, ccmD, ccmE, dsbE, NUW86_00165, NUW86_00290, NUW86_00295, NUW86_00420, NUW86_00475, NUW86_00690, NUW86_00740, NUW86_00835, NUW86_00845, NUW86_00855, araD, ulaD, NUW86_00935, NUW86_01005, NUW86_01010, tag, NUW86_01065, NUW86_01070, NUW86_01080, NUW86_01180, NUW86_01190, NUW86_01195, NUW86_01445, NUW86_01450, NUW86_01470, ugpQ, NUW86_01655, NUW86_01695, NUW86_01900, NUW86_01905, NUW86_01945, NUW86_02225, NUW86_02290, NUW86_02340, panF, NUW86_02390, rng, NUW86_02480, NUW86_02490, NUW86_02530, NUW86_02585, NUW86_02790, NUW86_02870, NUW86_02930, NUW86_02935, NUW86_02975, NUW86_03115, NUW86_03135, NUW86_03150, NUW86_03320, nudF, NUW86_03345, NUW86_03360, NUW86_03395, NUW86_03480, NUW86_03510, hybA, hybB, hybC, hybE, hypA, NUW86_03530, NUW86_03535, NUW86_03600, NUW86_03825, NUW86_03905, NUW86_04095, NUW86_04340, rlmM, NUW86_04440, gudP, NUW86_04535, NUW86_04625, ispD, ispF, surE, truD, NUW86_04670, NUW86_04675, NUW86_04680, NUW86_04690, NUW86_04695, NUW86_04705, NUW86_04710, NUW86_04925, orgA, orgB, prgJ, prgK, NUW86_05015, NUW86_05035, hycF, hycG, hycI, NUW86_05255, NUW86_05390, NUW86_05430, NUW86_05435, NUW86_05440, NUW86_05450, NUW86_05455, NUW86_05530, NUW86_05535, NUW86_05765, pssA, NUW86_05785, NUW86_05920, NUW86_05925, NUW86_06015, NUW86_06020, NUW86_06025, NUW86_06030, NUW86_06035, NUW86_06080, NUW86_06085, NUW86_06090, NUW86_06105, NUW86_06110, NUW86_06115, NUW86_06125, NUW86_06130, NUW86_06135, NUW86_06245, NUW86_06250, NUW86_06340, NUW86_06345, NUW86_06350, NUW86_06355, NUW86_06360, NUW86_06430, NUW86_06435, NUW86_06440, NUW86_06450, NUW86_06455, NUW86_06460, NUW86_06545, NUW86_06555, NUW86_06565, NUW86_06640, NUW86_06760, NUW86_06905, NUW86_06940, NUW86_06985, NUW86_07110, NUW86_07190, NUW86_07320, NUW86_07380, NUW86_07390, NUW86_07395, NUW86_07405, NUW86_07575, NUW86_07590, truA, NUW86_07635, NUW86_07675, hisM, NUW86_07730, NUW86_07735, NUW86_07740, NUW86_07775, NUW86_07870, NUW86_07875, NUW86_08180, NUW86_08195,

		NUW86_08205, ccmD, ccmE, dsbE, NUW86_08265, NUW86_08350, NUW86_08380, NUW86_08415, NUW86_08465, NUW86_08635, NUW86_08640, NUW86_08645, NUW86_08740, NUW86_08745, NUW86_08750, NUW86_08810, baeR, baeS, mdtC, NUW86_08845, NUW86_08925, cpsB, wcaI, NUW86_08995, rfbF, NUW86_09015, NUW86_09020, NUW86_09030, NUW86_09040, rfbK, NUW86_09185, NUW86_09305, cbiB, NUW86_09315, NUW86_09325, NUW86_09330, NUW86_09335, NUW86_09345, NUW86_09350, cbiD, cbiG, NUW86_09645, NUW86_09650, NUW86_09940, NUW86_09945, NUW86_10020, NUW86_10025, NUW86_10030, NUW86_10045, NUW86_10050, NUW86_10055, NUW86_10160, NUW86_10175, NUW86_10305, NUW86_10605, NUW86_10665, NUW86_10850, NUW86_10940, NUW86_11020, NUW86_11070, NUW86_11110, NUW86_11150, NUW86_11200, hyaB, NUW86_11310, narH, narI, narJ, NUW86_11340, NUW86_11430, oppF, NUW86_11475, NUW86_11535, trpD, NUW86_11565, cobO, NUW86_11645, NUW86_11785, NUW86_12065, NUW86_12070, NUW86_12180, NUW86_12230, NUW86_12305, narH, narI, narW, NUW86_12325, NUW86_12385, NUW86_12470, NUW86_12475, NUW86_12510, cybH, NUW86_12530, hybD, hypC, NUW86_12565, glsB, NUW86_12590, NUW86_12665, NUW86_12940, nth, NUW86_13000, NUW86_13005, NUW86_13015, NUW86_13085, NUW86_13095, NUW86_13165, ssaK, ssaL, NUW86_13255, NUW86_13340, NUW86_13375, NUW86_13405, NUW86_13435, NUW86_13450, NUW86_13705, astA, astB, astD, astE, NUW86_13785, NUW86_13935, NUW86_13940, NUW86_13945, NUW86_13950, NUW86_13955, NUW86_14075, NUW86_14080, NUW86_14090, NUW86_14095, NUW86_14100, NUW86_14170, NUW86_14175, NUW86_14180, NUW86_14185, NUW86_14190, NUW86_14285, NUW86_14290, NUW86_14740, NUW86_14780, NUW86_14785, NUW86_14820, ymdB, NUW86_14930, NUW86_15045, NUW86_15320, NUW86_15325, NUW86_15330, NUW86_15340, NUW86_15345, NUW86_15350, NUW86_15355, NUW86_15375, NUW86_15380, NUW86_15385, NUW86_15390, NUW86_15420, NUW86_15425, NUW86_15430, NUW86_15435, NUW86_15440, NUW86_15445, NUW86_15495, NUW86_15500, NUW86_15540, NUW86_15545, NUW86_15570, NUW86_15575, NUW86_15630, NUW86_15685, NUW86_15840, infA, NUW86_15855, NUW86_15870, NUW86_15945, NUW86_16070, NUW86_16075, NUW86_16080, NUW86_16085, NUW86_16090, NUW86_16155, NUW86_16160, NUW86_16350, NUW86_16355, cecR, hlyD, NUW86_16360,

		NUW86_16370, NUW86_16380, clsB, NUW86_16600, NUW86_16635, NUW86_16835, NUW86_16865, NUW86_16900, NUW86_17115, ybeY, NUW86_17435, NUW86_17440, NUW86_17525, entF, NUW86_17585, NUW86_17610, NUW86_17690, NUW86_17695, NUW86_17735, NUW86_17815, NUW86_17860, NUW86_17955, NUW86_17960, NUW86_18035, recR, NUW86_18155, NUW86_18160, NUW86_18255, NUW86_18260, cyoB, NUW86_18270, NUW86_18275, NUW86_18400, NUW86_18530, NUW86_18640, NUW86_18755, NUW86_18760, NUW86_18840, NUW86_19030, NUW86_19040, NUW86_19045, tssM, NUW86_19060, NUW86_19085, NUW86_19090, NUW86_19485, NUW86_19495, stfF, stfH, NUW86_19505, NUW86_19590, NUW86_19670, NUW86_19675, NUW86_19680, NUW86_19780, NUW86_19840, murC, NUW86_20210, NUW86_20220, NUW86_20305, NUW86_20355, NUW86_20365, bcfG, NUW86_20610, NUW86_20665, NUW86_20670, NUW86_20710, rimI, NUW86_20735, NUW86_20760, bglJ, NUW86_20800, NUW86_20805, NUW86_20810, NUW86_20825, NUW86_20875, hsdM, NUW86_20915, NUW86_21030, NUW86_21035, pglX, NUW86_21070, NUW86_21125, holC, NUW86_21130, NUW86_21155, argF, NUW86_21185, NUW86_21265, NUW86_21285, NUW86_21320, NUW86_21515, NUW86_21555, ulaE, NUW86_21615, NUW86_21850, NUW86_21915, NUW86_22085, NUW86_22145, NUW86_22150, NUW86_22160, NUW86_22215, siiA, siiC, siiD, NUW86_22375, NUW86_22380, NUW86_22385, NUW86_22415, NUW86_22425, NUW86_22430, NUW86_22435, NUW86_22440, NUW86_22495, NUW86_22500, NUW86_22505, NUW86_22515, NUW86_22520, NUW86_22525, NUW86_22565, NUW86_22580, NUW86_22700, thiC, thiE, thiS, NUW86_22925, NUW86_22930, NUW86_22955, NUW86_23065, NUW86_23160, NUW86_23165, NUW86_23175, NUW86_23295, NUW86_23300, NUW86_23435, NUW86_23565, NUW86_23765, dapF, xerC, yigB, NUW86_23875, rffM, wzxE, wzyE, NUW86_23895, rffC, NUW86_24035, NUW86_24065, NUW86_24185, NUW86_24205, oadA, oadA, odhB, ompA, ompS1, ompX, oppA, otsA, otsB, pal, panB, parC, pckA, pduA, pduB, pduC, pduD, pduE, pduG, pduH, pduK, pduL, pduM, pduN, pduO, pduP, pdxA, rsmA, surA, pdxB, pepD, pflA, pflB, pflB, pgi, pgk, pgpA, thiL, pgsA, pgtA, pgtB, pgtC, pheA, pheP, pheS, pheT, phnT, phnV, phoE, phoU, pitA, plaP, yoeI, plsB, plsC, plsX, pmbA, pmrB, pnp, pntB, potA, potB, potD, potH, potI, poxB, ppc, pphB, ppiA, ppiB, ppk1, pqiB, pqiC, prfA, prmC, sirB2, prfB, prfC, prgH, prlC, prmB, proB, proP, prpR, pspA, pspF, pstA, pstC, pstB, pstS, pta, ptrB, ptsG, ptsH, ptsI, ptsP, purB, purF, purH, purL, purM, purN, putA, putP,

		pykF, pyrD, pyrF, yciH, pyrG, qseE, radA, rapA, rarA, ratA, rbsB, rcsC, recA, recG, recJ, relA, rep, rfaC, rfaF, rfbA, rffG, wecB, wecC, rhaA, rhaB, rhaS, rhlE, ribA, ribF, ridA, rlmB, rlmI, rna, rne, rnhA, rnm, rnpB, robA, rph, rplA, rplB, rplC, rplD, rplW, rplF, rplJ, rplL, rplO, rplQ, rplR, rplV, rpmB, rpmD, rpoB, rpoC, rpoD, rpoE, rseD, rppH, rpsA, rpsB, rpsD, rpsG, rpsH, rpsJ, rpsK, rpsM, rpsO, rpsT, rseA, rseB, rseC, rsmE, rspA, rsuA, ruvA, sadA, sapA, sapB, sapC, sapD, sapF, sbcB, sbcC, sbcD, sdaB, sdaC, sdhA, sdhC, sdhD, secA, secF, secG, secY, selD, serA, setB, sgrR, shdA, siiE, siiF, sipA, sitA, sitB, sitC, sitD, slrP, sltY, slyD, sodA, solA, sopA, sopD, speB, speD, speE, srmB, ssb1, sseA, sseB, sspH2, ssrA, stbD, stbE, sthA, stjC, sucA, sucC, sucD, suhB, tamA, tamB, tatB, tatE, tcyL, yecC, tdcC, tdh, tgt, thiB, thiP, thiQ, thiI, thpR, thrA, thrB, thrC, thrS, thyA, tkt, tkt, tmcA, tnpA, tolA, tolB, tolC, topA, topB, torS, tpiA, treY, treZ, trmA, trxB, tsf, tssH, tuf, tuf, tus, typA, tyrA, tyrB, tyrR, tyrS, ubiA, ubiC, ubiD, ugpB, uhpA, uhpB, uhpT, ushA, uvrA, uvrB, uvrC, uvrY, wzz(fepE), wzzE, xanP, xylA, yajC, ybeD, ybiO, yccA, yccX, ycdR, ychH, ydgT, yeaG, yeaR, yejF, ygiD, ygjG, yhaJ, yhdP, yiaK, yicI, yidC, yieH, yjiA, yoaE, yvcK, zapA, zapE, zirT, znuA, znuB, znuC, zraR, zraS, zwf
Spark PFW	**27 Genes/ Loci:** *araB, atpA, ccmA, ccmB, dnaA, dnaE, fdhE, fdoI, fdxH, NUW86_00545, NUW86_00875, NUW86_02255, NUW86_05725, NUW86_08340, NUW86_15095, NUW86_16360, NUW86_19240, NUW86_21055, NUW86_22625, NUW86_22845, NUW86_24060, ppnN, rho, rseP, tcuA, tcuB, wecA*	**1304 Genes/Loci**: *aas, aat, accA, accB, accC, aceA, aceB, aceE, aceF, aceK, ackA, acnB, acpP, acpS, pdxJ, acrB, acrD, acs, adeP, adhE, adk, ahpC, alaC, alaS, aldB, alkB, allD, amiA, ampG, ampH, amyA, ansA, ansB, ansP, apt, araC, araE, arcB, argH, argR, argS, argT, arnA, arnC, arnD, arnE, arnF, arnT, aroA, aroB, aroG, aroK, artJ, artM, artQ, artP, asd, asmA, asnB, asnS, aspS, atpD, azoR, bamA, bamC, bamD, bcsB, bcsC, bcsZ, bepA, bioA, bioB, bioC, bioD, bioF, bioH, birA, murB, brxL, btsT, btuF, mtnN, cadA, cadB, caiF, caiT, can, carA, carB, chaA, chbG, cheB, cheR, cirA, citC, citD, citE, citD, citE, citF, citG, citX, citS, clcA, clpB, clpP, cmoM, mukB, mukE, mukF, cnoX, coaA, copA, corA, cpdA, yqiA, cpoB, cpsG, cpxA, cpxR, cpxP, creB, creC, creD, crp, csdA, csdE, csiE, cspE, cspE, cstA, cueP, cueR, cybC, cycA, cydA, cydB, cysA, cysP, cysT, cysW, cysD, cysE, cysG, cysH, cysM, cysS, dacA, dacD, dadX, dam, damX, dapD, dapE, dbpA, dcuB, dcuC, ddlA, deaD, yrbN, degP, deoA, deoB, der, dinG, dld, dmsA, dmsB, dnaG, dnaJ, dnaK, dnaN, dnaX, dpiA, dpiB, dppB, dppC, dppD, dppF, dsbA, dsdA, dsdX, dtpA, dtpB, dtpD, dusB, dusC, eco, efp, emrD, eno, entE, envC, epmA, era, rnc, eutA, eutH, eutB, eutC, eutR, fabA, fabB, fabF, fabH, fadB, fbaA, fbaB, fbp, fdhD, fdnG, fdnG, feoB, fepC, fepD, fepG, fes, fetA, fetB,*

		ffh, fhuB, fis, flgG, fliE, fliF, fliG, fliH, fliI, fliM, fliN, fliO, fliP, fliR, fliS, fliT, fnr, folD, folK, pcnB, fraB, frdA, frdB, fruB, fruK, fsa, ftsA, ftsQ, ftsH, ftsI, mraY, murE, murF, ftsK, ftsL, rsmH, ftsN, ftsW, murD, murG, ftsZ, fucA, fumB, fusA, gabD, gabT, galE, galK, galM, galP, galR, galT, gapA, garD, garR, gatD, gcvA, gcvP, gcvT, gdhA, ggt, glgA, glgC, glgB, glgX, glgP, glmS, glmU, glnA, glnE, glnG, glnH, glnL, glpE, glpK, glpX, gltB, gltD, gltJ, gltK, gltL, gltX, glxR, glyQ, glyS, gndA, gntK, gntU, gntR, gntT, golT, gpsA, secB, grcA, greA, gshA, gsiD, gspE, hofC, guaA, guaB, gudD, gutQ, srlR, gyrA, gyrB, hcp, helD, hemC, hemD, hemE, hemH, hemL, hemN, hflD, hflK, hflX, hfq, hilC, hisA, hisB, hisC, hisD, hisF, hisH, hisIE, hisS, hmpA, hmsP, hns, holB, tmk, yceG, hpaD, hpaE, hpaG, hpaI, hpt, hrpA, hrpB, hscA, hsdR, htpG, htpX, hutG, hutI, hutH, hycB, hycC, hycE, iaaA, ibpA, icd, ihfA, ileS, lspA, ilvA, ilvB, ilvC, ilvD, ilvE, ilvG, ilvM, ilvN, infB, infC, invC, spaK, spaM, spaN, spaO, spaP, iolB, iolC, iolE, iolT1, iroC, iscS, ispA, ispG, katE, katG, kdpB, kdsC, lptA, lptC, kup, ldcC, ldtA, ldtB, ldtD, lepA, leuA, leuB, leuC, leuE, leuS, lipA, livF, livG, livH, livM, livJ, livK, lldD, lldP, lldR, lolC, lolD, lolE, lon, lpdA, lplA, lplT, lptD, lpxA, lpxB, rnhB, lpxC, lpxD, lsrA, lsrC, lsrD, lsrB, lsrK, lysC, lysP, lysS, maa, maeB, malF, malG, malK, malP, malQ, malT, manA, manX, mazG, mdh, mdoG, mdoH, mdsA, mdsB, mdtA, mdtB, mdtD, mdtQ, mepS, metA, metE, metF, metG, metH, metK, metL, metQ, mfd, mglA, mglB, mgtA, miaA, mutL, minD, minE, mioC, mlaB, mlaC, mlaE, mltB, mltD, mnmA, mnmE, mnmG, moaA, moaB, moaE, motA, motB, mraZ, mrdA, mrdB, mreB, mreC, mtlA, mug, mutM, nadA, nadB, nadC, nadE, nadR, nagA, napG, napH, nepI, nfuA, nhaA, nifJ, nirB, nirD, nirC, nlpE, nlpI, nnr, tsaE, nrdA, nrdB, yfaE, nrdD, nrdR, nrfB, nrfC, nrfD, nrfEF, nrfG, nudC, nuoA, nuoC, nuoG, nuoH, nuoI, nuoJ, nuoK, nuoL, nuoN, nupC, nusA, nusG, NUW86_00030, NUW86_00050, NUW86_00055, dgoD, dgoR, NUW86_00065, NUW86_00115, NUW86_00130, NUW86_00140, ccmD, ccmE, dsbE, NUW86_00240, NUW86_00360, NUW86_00365, NUW86_00375, dgaE, NUW86_00385, NUW86_00435, NUW86_00475, NUW86_00775, NUW86_00910, NUW86_00965, NUW86_00970, NUW86_01005, NUW86_01010, tag, NUW86_01020, NUW86_01070, NUW86_01080,

		NUW86_01175, NUW86_01195, NUW86_01215, NUW86_01220, NUW86_01330, NUW86_01355, NUW86_01365, NUW86_01470, ugpQ, NUW86_01500, NUW86_01520, NUW86_01580, NUW86_01585, NUW86_01590, NUW86_01715, NUW86_01775, NUW86_01780, NUW86_01785, NUW86_01790, hofQ, NUW86_01840, tnpA, NUW86_01945, NUW86_02245, NUW86_02265, NUW86_02290, NUW86_02295, NUW86_02465, NUW86_02490, NUW86_02530, NUW86_02600, NUW86_02875, NUW86_02880, NUW86_02885, NUW86_02890, NUW86_02895, NUW86_02905, NUW86_02930, NUW86_02935, NUW86_03040, NUW86_03065, NUW86_03100, NUW86_03110, NUW86_03150, NUW86_03155, NUW86_03160, NUW86_03300, NUW86_03305, NUW86_03320, nudF, NUW86_03400, NUW86_03455, NUW86_03550, NUW86_03575, NUW86_03580, NUW86_03595, NUW86_03610, NUW86_03615, NUW86_03655, NUW86_03735, yggU, NUW86_03740, NUW86_03760, ruvX, NUW86_03855, NUW86_03865, NUW86_03870, NUW86_03875, NUW86_03905, NUW86_03935, NUW86_03985, NUW86_04185, NUW86_04215, NUW86_04340, rlmM, NUW86_04440, gudP, NUW86_04535, NUW86_04625, ispD, ispF, surE, truD, NUW86_04670, NUW86_04675, NUW86_04680, NUW86_04685, NUW86_04690, NUW86_04695, NUW86_04705, NUW86_04710, NUW86_04745, NUW86_04925, orgA, orgB, prgJ, prgK, NUW86_05015, NUW86_05035, hycF, hycG, hycI, NUW86_05100, srlA, NUW86_05190, NUW86_05335, NUW86_05385, NUW86_05390, NUW86_05495, NUW86_05535, NUW86_05560, NUW86_05570, NUW86_05670, dgcN, NUW86_05735, NUW86_05785, NUW86_05865, NUW86_05870, NUW86_05920, NUW86_05925, NUW86_05960, NUW86_05965, NUW86_05970, NUW86_06015, NUW86_06020, NUW86_06025, NUW86_06030, NUW86_06035, NUW86_06075, NUW86_06080, NUW86_06085, NUW86_06090, NUW86_06105, NUW86_06110, NUW86_06115, NUW86_06125, NUW86_06130, NUW86_06135, NUW86_06190, NUW86_06195, NUW86_06245, NUW86_06250, NUW86_06340, NUW86_06345, NUW86_06350, NUW86_06355, NUW86_06360, NUW86_06430, NUW86_06435, NUW86_06440, NUW86_06450, NUW86_06455,

		NUW86_06460, NUW86_06565, NUW86_06640, NUW86_06645, NUW86_06760, NUW86_06795, NUW86_06895, NUW86_06955, NUW86_06985, NUW86_07110, NUW86_07305, NUW86_07380, NUW86_07390, NUW86_07470, NUW86_07485, NUW86_07640, NUW86_07765, NUW86_07770, NUW86_07875, NUW86_07995, NUW86_08005, NUW86_08045, NUW86_08105, NUW86_08125, NUW86_08245, NUW86_08295, NUW86_08310, NUW86_08355, NUW86_08365, NUW86_08370, NUW86_08445, NUW86_08475, NUW86_08495, NUW86_08555, NUW86_08635, NUW86_08640, NUW86_08645, NUW86_08660, btsR, NUW86_08810, baeR, baeS, mdtC, NUW86_08845, NUW86_08870, NUW86_08925, cpsB, wcaI, NUW86_09015, NUW86_09185, NUW86_09305, cbiB, NUW86_09315, NUW86_09325, NUW86_09330, NUW86_09335, NUW86_09345, NUW86_09350, cbiD, cbiG, NUW86_09380, NUW86_09385, cobS, cobU, NUW86_09480, NUW86_09645, NUW86_09650, NUW86_09895, NUW86_09900, NUW86_09905, NUW86_09940, NUW86_09945, NUW86_09975, NUW86_09980, NUW86_09985, NUW86_10110, NUW86_10265, NUW86_10435, NUW86_10580, NUW86_10605, NUW86_10760, NUW86_10815, NUW86_10970, NUW86_11110, NUW86_11115, NUW86_11125, NUW86_11200, hyaB, NUW86_11310, narH, narI, narJ, NUW86_11430, oppF, NUW86_11475, NUW86_11505, NUW86_11790, NUW86_11825, NUW86_11850, NUW86_12000, NUW86_12060, NUW86_12065, NUW86_12070, NUW86_12075, NUW86_12095, aac(6'), NUW86_12220, srfC, NUW86_12285, NUW86_12305, narH, narI, narW, NUW86_12420, NUW86_12485, NUW86_12490, NUW86_12500, NUW86_12530, hybD, hypC, NUW86_12535, NUW86_12540, NUW86_12660, NUW86_12665, NUW86_12705, NUW86_12770, NUW86_12815, ydgH, NUW86_13705, astA, astB, astD, astE, NUW86_13835, NUW86_13865, NUW86_13885, NUW86_13985, NUW86_14020, NUW86_14075, NUW86_14080, NUW86_14090, NUW86_14095, NUW86_14100, NUW86_14115, NUW86_14120, NUW86_14125, NUW86_14170, NUW86_14175, NUW86_14180, NUW86_14185, NUW86_14190, NUW86_14285, NUW86_14290, NUW86_14410, NUW86_14765, NUW86_14880, NUW86_14920, NUW86_14930,

		NUW86_15045, NUW86_15090, hprR, NUW86_15175, NUW86_15195, NUW86_15325, NUW86_15330, NUW86_15340, NUW86_15345, NUW86_15350, NUW86_15355, NUW86_15375, NUW86_15380, NUW86_15420, NUW86_15425, NUW86_15430, NUW86_15435, NUW86_15440, NUW86_15445, NUW86_15495, NUW86_15500, NUW86_15570, NUW86_15575, NUW86_15635, NUW86_15685, NUW86_15775, NUW86_15945, NUW86_15955, NUW86_15960, NUW86_16000, NUW86_16055, nfsA, NUW86_16070, NUW86_16090, NUW86_16220, NUW86_16230, NUW86_16245, NUW86_16270, NUW86_16350, NUW86_16355, cecR, hlyD, NUW86_16370, NUW86_16380, clsB, NUW86_16495, NUW86_16510, NUW86_16585, NUW86_16590, NUW86_16820, NUW86_16825, NUW86_16850, glf, NUW86_16865, NUW86_16910, NUW86_16960, NUW86_16990, fur, NUW86_17115, ybeY, NUW86_17180, NUW86_17185, NUW86_17305, NUW86_17395, NUW86_17400, NUW86_17405, NUW86_17410, NUW86_17445, NUW86_17450, NUW86_17475, entA, NUW86_17525, entF, NUW86_17535, NUW86_17545, NUW86_17595, NUW86_17600, NUW86_17765, NUW86_17770, NUW86_17775, NUW86_17805, NUW86_17860, NUW86_17900, sfbB, NUW86_17915, NUW86_17920, NUW86_17925, NUW86_17935, NUW86_17955, NUW86_17960, NUW86_17995, NUW86_18035, recR, NUW86_18105, NUW86_18120, NUW86_18180, NUW86_18255, NUW86_18260, cyoB, NUW86_18360, NUW86_18415, NUW86_18600, NUW86_18615, NUW86_18665, cydB, NUW86_18685, NUW86_18750, NUW86_18805, NUW86_18810, NUW86_18960, NUW86_19030, NUW86_19225, NUW86_19255, metN, NUW86_19295, rof, NUW86_19620, NUW86_19625, NUW86_19640, NUW86_19740, NUW86_19745, NUW86_19840, murC, NUW86_19970, NUW86_20090, NUW86_20220, NUW86_20340, NUW86_20355, NUW86_20465, NUW86_20515, NUW86_20535, NUW86_20625, NUW86_20665, NUW86_20670, NUW86_20710, rimI, NUW86_20765, NUW86_20770, NUW86_20820, NUW86_20825, NUW86_20900, NUW86_20980, NUW86_20995, NUW86_21030, NUW86_21035, pglX, NUW86_21125, holC, NUW86_21155, argF,

		NUW86_21270, NUW86_21275, NUW86_21280, NUW86_21290, NUW86_21320, NUW86_21460, NUW86_21470, NUW86_21555, ulaE, NUW86_21630, NUW86_21635, NUW86_21640, NUW86_21645, NUW86_21740, NUW86_21880, cutA, NUW86_21920, NUW86_21925, NUW86_21970, dmsB, NUW86_21980, NUW86_22085, NUW86_22135, actP, NUW86_22160, NUW86_22275, NUW86_22290, NUW86_22345, NUW86_22425, NUW86_22430, NUW86_22435, NUW86_22440, NUW86_22515, NUW86_22520, NUW86_22565, NUW86_22615, NUW86_22700, thiC, thiE, thiS, NUW86_22830, NUW86_22955, NUW86_22975, NUW86_23085, NUW86_23235, NUW86_23240, NUW86_23350, NUW86_23355, NUW86_23360, NUW86_23450, NUW86_23455, NUW86_23460, NUW86_23550, NUW86_23565, NUW86_23580, pepQ, NUW86_23685, NUW86_23735, NUW86_23765, dapF, xerC, yigB, NUW86_23780, NUW86_23785, NUW86_23790, NUW86_23895, rffC, NUW86_23975, NUW86_23980, NUW86_24050, NUW86_24215, NUW86_24270, yidZ, NUW86_24285, oadA, oadA, oadA, odhB, ompA, ompS1, oppA, oxyR, pabA, pabB, pal, panC, panE, yajL, parC, parE, patD, pckA, pdhR, pduA, pduB, pduE, pduG, pduH, pduQ, pduS, pduV, pduW, pdxA, rsmA, surA, pdxH, pepA, pepP, ubiH, pfkA, pflB, pflB, pgi, pgk, pgl, pglZ, pgm, pgtA, pgtB, pgtC, pheA, pheS, phnT, phoP, phoQ, phoU, pldB, plsB, plsX, pmbA, pncC, pnp, pntB, polB, potE, speF, potF, potH, potI, ppc, ppiA, ppiD, ppk1, ppsA, ppx, prc, prfA, prmC, sirB2, prfB, prfC, proC, proS, proV, proW, proY, pspA, pstB, pta, ptrA, recB, recD, ptrB, ptsG, ptsG, ptsI, ptsJ, punC, purA, purB, purE, purK, purL, putA, putP, pykF, pyrF, yciH, pyrG, pyrH, queA, radA, rapA, rarA, rarD, ratB, ravA, viaA, rbfA, truB, rbsA, rbsC, rcsB, rcsC, rcsD, recG, recQ, relA, rfaD, rhaA, rhaB, rhaS, rhaT, rhlB, rhlE, ribF, rimO, ripC, ripR, rlmB, rlmD, rlmE, rlmKL, rluB, rluD, yfiH, rluF, rmf, rnpA, yidD, rnpB, rnr, rplA, rplB, rplC, rplE, rplI, rplJ, rplK, rplM, rplN, rplO, rplP, rpmC, rpsQ, rplQ, rplR, rplV, rpmB, rpoA, rpoB, rpoC, rpoH, rpoN, rpoS, rpsA, rpsD, rpsE, rpsF, rpsG, rpsH, rpsK, rpsM, rpsP, rraA, rrf, rrf, rseA, rseB, rseC, rsmE, rsmI, rstB, rsxD, rtcA, rtcR, sapA, sapB, sapC, sapD, sapF, sbcC, sbcD, sbmA, sdaA, sdaB, sdaC, sdhA, sdhC, sdhD, sdhB, secA, secD,

		secF, secY, selD, serA, shdA, sifA, siiF, sitA, sitB, sitC, sitD, sltY, smpB, smvA, sodA, sodB, speA, speG, spoT, srfJ, srlD, sseD, sspA, ssrA, sstT, stbD, stbE, stdB, stfD, sthA, sthE, sucA, sucC, sucD, sufA, sufC, sufD, sufS, tadA, tal, tamA, tamB, tatC, tatD, tgt, thiG, thiH, thiI, thrA, thyA, tig, tkt, tnpA, tnpA, tolB, tonB, topB, torA, torC, torD, tpiA, treB, trmD, trpA, trpB, tsf, tsr, tssK, tssL, ttcA, ttdA, ttdB, tuf, typA, tyrB, ubiF, ubiK, uhpA, uhpB, uhpT, ulaA, ulaB, ulaC, ulaG, uraA, ushA, uvrA, uvrB, uvrC, uvrY, uvrD, vapB, vapC, waaA, wcaM, wzz(fepE), xanP, xthA, yacL, ybiO, ycjG, yeaG, yegQ, yejM, yfhb, ygbI, ygfZ
Glow PFW	**33 Genes/Loci:** *araB, atpA, carB, ccmA, ccmB, cysI, cysJ, dnaA, dnaE, fdhE, fdoI, fdxH, groL, NUW86_00545, NUW86_00875, NUW86_02255, NUW86_05725, NUW86_08340, NUW86_15095, NUW86_16360, NUW86_19240, NUW86_21055, NUW86_22625, NUW86_22845, NUW86_23565, NUW86_24060, ppnN, rho, rpoC, rseP, tcuA, tcuB, wecA*	**137 Genes/ Loci:** *acpS, araD, argG, asnB, atpE, bsmA, cgtA, clcA, clpX, crr, ccmD, ccmE, cspE, csrA, cysA, cysP, cysT, cysW, cysK, cysS, dacB, ddlA, dnaK, dsbE, fdhD, fldA, fljA, ftsZ, glgP, glrR, qseG, gltB, gltD, glyS, guaB, hybG, hycE, ibpA, kup, ldcC, lon, lplA, lpxD, lysM, manY, metB, metR, norV, norW, NUW86_00845, NUW86_00855, NUW86_01190, NUW86_01225, NUW86_02265, NUW86_03110, NUW86_05460, NUW86_05735, NUW86_06015, NUW86_06020, NUW86_06025, NUW86_06030, NUW86_06035, NUW86_06105, NUW86_06110, NUW86_06440, NUW86_06640, NUW86_06655, NUW86_06660, NUW86_08180, NUW86_08195, NUW86_08205, NUW86_08785, NUW86_09415, NUW86_10395, NUW86_13455, NUW86_13460, NUW86_14090, NUW86_14170, NUW86_14175, NUW86_14180, NUW86_14185, NUW86_14190, NUW86_16070, NUW86_16600, NUW86_19225, NUW86_20220, NUW86_20240, NUW86_21740, NUW86_22290, NUW86_22615, NUW86_22830, NUW86_23550, NUW86_23875, NUW86_24050, oadA, ppc, pdxJ, ppiB, prfC, proY, prpR, pta, rarA, rffM, rhaT, rluC, rnpB, rplB, rplD, rplW, rplU, rpmA, rpoA, rpsB, rpsC, rrf, sdhB, secY, setB, sopB, ssb1, ssrS, sucC, sucD, thrS, topA, tuf, tyrS, ubiD, ubiF, udp, ulaD, uvrD, waaZ, wzxE, wzyE, yeaG*
Ultraviolet LED	**34 Genes/ Loci:** *araB, atpA, carB, ccmA, ccmB, cysI, cysJ, dnaA, dnaE, fdhE, fdoI, fdxH, groL, NUW86_00545, NUW86_00875, NUW86_02255, NUW86_02265, NUW86_08340, NUW86_15095, NUW86_16360, NUW86_19240, NUW86_21055, NUW86_22615, NUW86_22625, NUW86_22830, NUW86_23550, NUW86_24050,*	**40 Genes/Loci:** *adhE, adiC, dnaX, dppD, dppF, fusA, gmk, hmsP, malE, mdh, nrdD, nuoJ, nuoK, nuoL, NUW86_01945, NUW86_02600, NUW86_04215, NUW86_05725, NUW86_05735, NUW86_06440, NUW86_11345, NUW86_14090, NUW86_16245, NUW86_16800, NUW86_19225, NUW86_22845, NUW86_23565, NUW86_23895, rffC, NUW86_23975, NUW86_23980, NUW86_24060, parC, phnW, ppc, rplB, rpoB, secY, tuf, yoaE*

	ppnN, rho, rpoC, rseP, tcuA, tcuB, wecA	
